# Neonates living with enterostomy following necrotising enterocolitis are at high risk of becoming severely underweight

**DOI:** 10.1007/s00431-019-03440-6

**Published:** 2019-09-14

**Authors:** Clara Chong, Jacqueline van Druten, Graham Briars, Simon Eaton, Paul Clarke, Thomas Tsang, Iain Yardley

**Affiliations:** 1 Department of Paediatric Surgery, Evelina Children’s Hospital, Westminster Bridge Rd, Lambeth, London, SE1 7EH UK; 2 Department of Paediatric Surgery, Norfolk and Norwich University Hospitals NHS Foundation Trust, Colney Lane, Norwich, NR4 7UY UK; 3 Department of Nutrition and Dietetics, Norfolk and Norwich University Hospitals NHS Foundation Trust, Norwich, Colney Lane, Norwich, NR4 7UY UK; 4 Department of Paediatric Gastroenterology, Norfolk and Norwich University Hospitals NHS Foundation Trust, Colney Lane, Norwich, NR4 7UY UK; 5 Norwich Medical School, University of East Anglia, Norwich, NR4 7TJ UK; 6 UCL Great Ormond Street Institute of Child Health, London, WC1N 1EH UK; 7 Neonatal Intensive Care Unit, Norfolk and Norwich University Hospitals NHS Foundation Trust, Colney Lane, Norwich, NR4 7UY UK; 8 Faculty of Life Sciences & Medicine, King’s College London, Strand, London, WC2R 2LS UK

**Keywords:** NEC, *z*-score, Stoma, Severe underweight, Growth failure, Intestinal failure

## Abstract

Necrotising enterocolitis (NEC) is often managed with a temporary enterostomy. Neonates with enterostomy are at risk of growth retardation during critical neurodevelopment. We examined their growth using *z*-score. We identified all patients with enterostomy from NEC in two neonatal surgical units (NSU) during January 2012–December 2016. Weight-for-age *z*-score was calculated at birth, stoma formation and closure, noting severely underweight as *z* < − 3. We compared those kept in NSU until stoma closure with those discharged to local units or home (LU/H) with a stoma. A total of 74 patients were included. By stoma closure, 66 (89%) had deteriorated in *z*-score with 31 (42%) being severely underweight. There was no difference in *z*-score at stoma closure between NSU and LU/H despite babies sent to LU/H having a more distal stoma, higher birth weight and gestational age. Babies in LU/H spent a much shorter period on parenteral nutrition while living with their stoma for longer, many needing readmission.

*Conclusion*: Growth failure is a common and severe problem in babies living with enterostomy following NEC. *z*-score allowed growth trajectory to be accounted for in nutrition prescription and timing of stoma closure. Care during this period should be focused on minimising harm.

**Table:** 

**What is Known:** • *Necrotising enterocolitis (NEC) is a life-threatening condition affecting predominately premature and very low birth weight neonates. Emergency treatment with temporary enterostomy often leads to growth failure.* • *There is no consensus on the optimal timing for stoma reversal, hence prolonging impact on growth during crucial developmental periods. Both malnutrition and surgical NEC are independently associated with poor neurodevelopment outcome.*	
**What is New:** • *Our study found growth in 89% of babies deteriorated while living with a stoma, with 42% having a weight-for-age z-score < − 3, meeting the WHO criteria of being severely underweight, despite judicial use of parenteral nutrition. Applying z-score to weight measurements will allow growth trajectory to be accounted for in clinical decisions, including nutrition prescription (both enteral and parenteral), and guide timing of stoma closure.* • *Surgeons who target stoma closure at a certain weight risk waiting for an indefinite period of time, during which babies’ growth may falter.*

**What is Known:**

• *Necrotising enterocolitis (NEC) is a life-threatening condition affecting predominately premature and very low birth weight neonates. Emergency treatment with temporary enterostomy often leads to growth failure.*

• *There is no consensus on the optimal timing for stoma reversal, hence prolonging impact on growth during crucial developmental periods. Both malnutrition and surgical NEC are independently associated with poor neurodevelopment outcome.*

**What is New:**

• *Our study found growth in 89% of babies deteriorated while living with a stoma, with 42% having a weight-for-age z-score < − 3, meeting the WHO criteria of being severely underweight, despite judicial use of parenteral nutrition. Applying z-score to weight measurements will allow growth trajectory to be accounted for in clinical decisions, including nutrition prescription (both enteral and parenteral), and guide timing of stoma closure.*

• *Surgeons who target stoma closure at a certain weight risk waiting for an indefinite period of time, during which babies’ growth may falter.*

## Introduction

Necrotising enterocolitis (NEC) is an ischaemic and inflammatory condition that affects the intestine of neonates, especially those born prematurely or with very low birth weight [5]. A recent UK surveillance study (2012–2013) showed that 80% (423/531) of babies with severe necrotising enterocolitis underwent laparotomy [4]. Surgical management of NEC often involves the formation of a temporary enterostomy with or without bowel resection [15]. Enterostomies in neonates are associated with a high risk of complications, up to 68% in some series [20], with complications being more common in extremely preterm neonates or those with extremely low birth weight (ELBW; < 1000 g) [1, 6]. Potential complications include gross electrolyte losses, poor weight gain, stomal prolapse, retraction, strictures and parastomal herniation [1, 6, 25]. Fluid and electrolyte losses from high output stomas can be life-threatening, especially in ELBW infants [1, 6, 16, 25]. Neonates with enterostomies therefore require careful management by an experienced multidisciplinary team, with meticulous attention paid to their nutritional status [6, 16, 27]. For this reason, neonates with enterostomies are often kept in neonatal surgical units (NSU) until stoma closure. Currently there is no clear consensus on the optimal timing for stoma closure [23, 30]. Some surgeons advocate early reversal, usually at 6 to 8 weeks after stoma formation, to mitigate the problems outlined above [29] while others prefer to wait until the baby is larger to offset the risks of surgery in very small babies [2, 3, 17]. Caring for babies in NSU until stoma closure has implications for both the wider healthcare system and for the family of the neonate who may be far from home, making discharging babies with an enterostomy to their local, non-surgical unit or home (LU/H) a common practice.

In this study, we aimed to examine the growth of neonates living with enterostomy following NEC from stoma formation until closure. We sought to compare growth in those cared for in NSU until stoma reversal with growth in those discharged to LU/H with their stoma in situ to determine whether discharge prior to stoma closure is a safe practice.

## Materials and methods

We identified all patients who underwent formation of an enterostomy following a diagnosis of NEC in two UK NSU over a 5-year period (January 2012–December 2016) using the nationwide electronic clinical data management system BadgerNet Neonatal (CleverMed Ltd., Edinburgh, UK). There are 29 NSU in the UK; the two NSU centres participating in the present study individually manage > 5000 live birth per annum, and each covers a catchment area population of more than 1 million. Exclusion criteria were death prior to stoma closure, unavailability of data around stoma closure and a diagnosis other than NEC associated with stoma creation. Demographic data including gender and gestational age were recorded. The site of the enterostomy (jejunostomy, ileostomy or colostomy) was determined from the operative records. Histology records were reviewed to confirm the diagnosis of NEC in babies who underwent intestinal resection. Weights at three time points were noted: birth, stoma formation and stoma closure. Weights were plotted using the UK-WHO close monitoring growth chart [28], and a weight-for-age *z*-score at each time point was calculated using LMS growth Excel add-on [21]. We used the WHO definition of a weight-for-age *z*-score of < − 3 to identify those babies who were severely underweight [7, 10, 14, 18]. A baby is considered to have faltering growth with a falling *z*-score over time.

Patients were analysed in two groups: those kept in the NSU for stoma closure during their index admission and those discharged to LU/H with a stoma in situ for planned closure on a subsequent admission. The primary outcome was growth expressed by weight-for-age *z*-score. Data were presented as median (range). Statistical analysis was undertaken using the Mann-Whitney *U* test for unpaired and Wilcoxon signed-rank test for paired data. Parametric data were analysed using a *t* test and marked with *.

The study was approved by the Evelina London Children’s Hospital audit department (registration number: 7693). The study was deemed a clinical audit/service evaluation and did not require formal research ethics approval under contemporaneous UK National Research Ethics Service guidelines.

## Results

A total of 109 neonates were identified as having undergone enterostomy formation for NEC during the study period. Thirty-five patients were excluded: 21 died prior to stoma closure; 10 had no electronic information on stoma closure; and 4 did not have a diagnosis of NEC on review of histology (1 volvulus, 3 spontaneous intestinal perforations). Seventy-four neonates were included, and postnatal age at stoma formation was 32 (26–46) weeks. Forty-six (62%) remained as in-patients in the NSU until their stoma was closed, and 28 (38%) were discharged to their LU/H with their stoma in situ. Babies kept in the NSU were of significantly lower birth weight and gestational age, and tended to have a more proximal stoma (Table 1). All 14 babies with a jejunostomy were kept in their NSU until their stoma was closed. Thirty-three (46%) patients received parenteral nutrition (PN) for > 90 days; all these were cared for in the NSU until stoma closure. Babies discharged to LU/H spent significantly shorter periods on PN but lived with their stoma for longer.

**Table 1:** Patient demographics

	NSU	LU/H	*P* value
*n*	46	28	
Birth weight (g)	865 (450 to 3085)	1175 (576 to 4320)	0.009
Gestation (weeks)	26 (23 to 39)	29 (24 to 39)	0.02
Birth *z*-score	− 0.6 (2.6 to − 3.6)	− 0.4 (2.4 to −3)	0.3
Day of life stoma formed (day)	26 (4 to 130)	12 (3 to 87)	0.0006
Postnatal age stoma formed (weeks)	32 (26 to 46)	31 (27 to 40)	*0.4
Stoma type, *n*			
Jejunostomy	14	–	
Ileostomy	32	25	
Colostomy	–	3	
Time to closure (weeks)	11 (3 to 21)	14 (7 to 67)	0.001
Postnatal age stoma closed (weeks)	43 (33 to 59)	46 (39 to 96)	0.001
Duration on parenteral nutrition (days)	103 (18 to 200)	27 (11 to 89)	< 0.00001
Fulfilled criteria of IF for being on PN for > 90 days, *n* (%)	33 (72)	0	

*t test applied for parametric data ; data are median (range) unless otherwise specified

### Growth as expressed by weight-for-age *z*-score

**Fig. 1:**
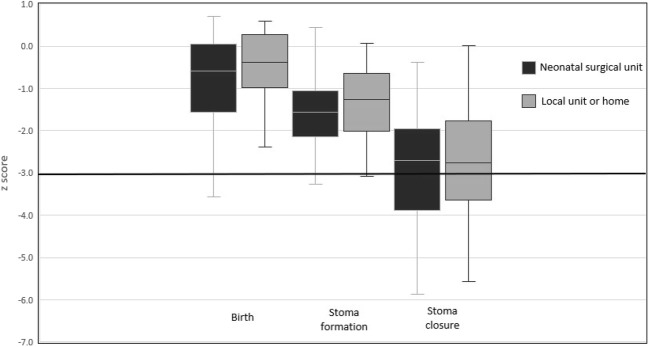
Box plot for *z*-score at birth, stoma formation and closure by location of care

**Fig. 2:**
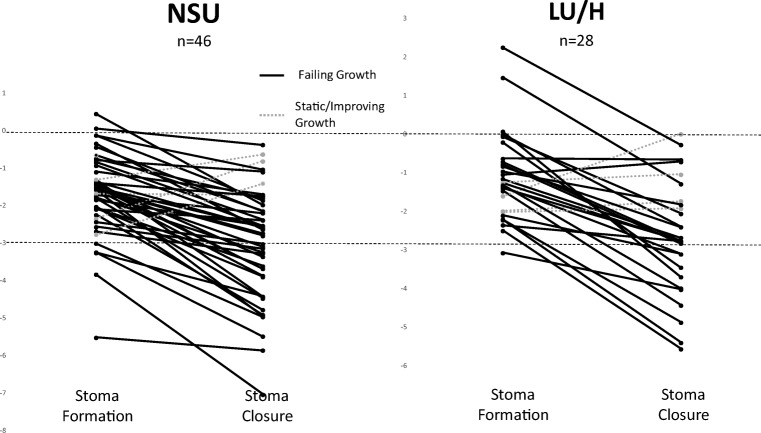
Line graph with individual patients’ growth trajectories

Restricted growth was common among neonates needing surgery for NEC, even prior to surgery (Fig. 1), reflected by 68 (92%) patients having a *z*-score below 0 at the time of their stoma formation (expected would be 50%). Growth was further retarded during the time they lived with the stoma. Stoma closure occurred at median postnatal age 44 (36–96) weeks. At the time of stoma closure, all but one baby had a *z*-score below zero and 31 (42%) had a *z*-score < − 3, therefore fulfilling the WHO classification of being severely underweight (Table 2) [7]. Given that for any individual baby the expected *z*-score at stoma closure is dependent upon their birth *z*-score, we also examined changes in *z*-score in individual babies (Fig. 2). Most babies (66/74, 89%) experienced a fall in their *z*-score between stoma formation and reversal, and this growth failure was highly significant (*P* < 0.00001, paired Wilcoxon signed-rank analysis).

**Table 2:** Weight and weight-for-age *z*-score at stoma formation and closure

	NSU	LU/H	*P* value
	*n* = 46	*n* = 28	
Weight at stoma formation (g)	1200 (550 to 3490)	1308 (576 to 4320)	0.4
*z*-score at stoma formation	− 1.6 (0.4 to − 5.5)	− 1.3 (0.1 to − 3.1)	0.05
Weight at stoma closure (g)	2598 (1100 to 4435)	3248 (1860 to 8760)	0.0005
*z*-score at stoma closure	− 2.7 (− 0.6 to − 7)	− 2.8 (0 to − 5.6)	0.5

Data shown are median (range)

When absolute weights were compared, those discharged to their LU/H weighed significantly more at stoma closure compared with their counterparts kept in the NSU. Although discharged babies waited longer before their enterostomies were reversed, with a median delay of 3 weeks for those in LU/H, when age was taken into consideration their growth failure was similarly severe, as demonstrated by the similar weight-for-age *z*-scores between the two groups. Of the babies discharged/transferred, 11 (39%) required emergency readmission, mainly due to failing to thrive secondary to high stoma output requiring central access for nutrition.

## Discussion

We found that significant faltering growth occurred in most babies (89%) with an enterostomy following NEC, regardless of the location of their care. Worryingly, 42% of babies met the criteria for being severely underweight at the time of stoma closure. This was true of babies discharged to their LU/H prior to stoma closure despite them being larger, more robust babies with more distal stomas. In addition, a significant proportion (39%) of those discharged to LU/H required emergency readmission due to fluid and electrolyte losses from their stoma.

Our results are in keeping with the findings of previous series demonstrating that neonates needing surgery for NEC are at high risk of growth restriction. Mansour et al. [18] studied weight *z*-scores in a specialist neonatal surgical unit and found that babies with a stoma, particularly an ileostomy, grew poorly. Similarly, Bethell et al. [6] demonstrated growth failure in babies with a stoma which resolved following closure.

### Monitoring growth using *z*-score

Many of our patients were born with a weight at the lower end of their gestational centile. Their weights rapidly plummeted below the 0.4th centile as they went through a period of catabolism caused by developing NEC and undergoing surgery, and therefore did not follow any lines on the centile chart. Their subsequent weight gain which dictated nutrition prescription while living with a stoma was only monitored using a daily weight chart without references for comparison. Converting absolute weight to weight-for-age *z*-scores facilitated early detection of growth failure, thus guiding subsequent nutritional management in the high-risk patient cohort [13].

### Intestinal failure

The definition of intestinal failure as ‘a reduction of gut function below the minimum necessary for the absorption of macronutrients, water and electrolytes, such that intravenous supplementation is required to maintain health and growth’ [8] can be applied to almost all patients in our study. The alternative and often used criterion of ‘dependent on PN for longer than 90 days’ was applicable to 72% of our NSU patients, in particular those with a proximal enterostomy. We would argue that those whose growth faltered drastically on enteral feed alone, especially those at LU/H, should have been on PN and would also qualify as intestinal failure (IF) cases. Intestinal failure, however, was not found to be a diagnosis associated with our patient cohort. Duro et al. [9] reported a 5-fold increased risk of developing PN-associated liver disease in neonates with a jejunostomy from NEC compared with those who underwent other surgical interventions in a multi-centre prospective study. Furthermore, a survey of PN prescription in all neonatal units in the UK demonstrated significant sub-optimal nutrition prescription practice as assessed against the European Society for Parenteral and Enteral Nutrition (ESPEN) guideline, leading to iatrogenic malnutrition [14]. For neonates with a stoma, earlier and proactive diagnosis of IF could mobilise resources, such as a dedicated multi-disciplinary nutritional support team which focused on meticulous nutrition optimisation and prevention of complications, for example liver disease and catheter-related sepsis [8].

### Neurodevelopment

The neonatal period is a crucial phase of brain growth and development [19, 22]. There are long established links between malnutrition and poor neurodevelopment [11, 12, 26] and there is mounting evidence of impaired neurodevelopment in undergoing surgery for NEC compared with their medically-managed counterparts [24]. A long-term follow-up study looking at neurodevelopmental outcomes in children who had undergone surgical treatment for NEC as neonates has demonstrated significant impairment in the total intelligence quotient and motor skills in those who received a stoma compared with those who underwent primary anastomosis, even when disease severity was taken into account [24]. This demonstrates that faltering growth while awaiting stoma reversal has long-term implications and so there must be a high degree of urgency for maintaining adequate nutrition during this crucial growth period.

Our study has the strength of having examined a large cohort of patients over several years in two large neonatal surgical centres. To our knowledge, it is the largest longitudinal cohort study to date of babies with a stoma following NEC and the first to apply the WHO definition of severely underweight to quantify growth failure in NEC. The use of a nationwide electronic clinical database made it possible to monitor the progress of babies after discharge, even in remote units, and our data capture was complete for all babies included in the cohort. Limitations include that this was a retrospective study, and confounding factors may have been at play, for example, changes in practice over time. There were significant demographic differences with smaller, more premature babies being less likely to be discharged with a stoma than their larger, more robust counterparts. We have also only used weight as a proxy for general wellbeing, and there may have been other significant differences in co-morbidities between the two groups that we have not measured. Also, we have not made any attempt to assess the less tangible benefits of care closer to home for those discharged to local units with a stoma, such as the psychological well-being of the wider family.

Our study has shown that growth failure is a common and severe problem in babies living with a stoma following NEC. Applying *z*-score to weight measurements will allow growth trajectory to be accounted for in clinical decisions, including nutrition prescription and timing of stoma closure. Surgeons who target stoma closure at a certain weight risk waiting for an indefinite period of time, during which babies’ growth may further falter. Further research is needed to better define the optimum time of enterostomy reversal following NEC. Given the relative rarity of the condition and the wide individual variability among these babies, this will require a coordinated, multicentre approach. Further studies are also needed on how nutrition and growth can be best maintained in babies with an enterostomy in situ.

## Abbreviations

ELBW: Extremely low birth weight
IF: Intestinal failure
LU/H: Local units or home
NEC: Necrotising enterocolitis
NSU: Neonatal surgical units
PN: Parenteral nutrition
